# Measuring the Electromagnetic Field of Human Subjects Using Induction Sensors and a Shielded Helmet Without the Need for a Shielded Room

**DOI:** 10.7759/cureus.24107

**Published:** 2022-04-13

**Authors:** James Brazdzionis, James Wiginton, Paras Savla, James Hung, Yongming Zhang, Dan E Miulli

**Affiliations:** 1 Neurosurgery, Riverside University Health System Medical Center, Moreno Valley, USA; 2 Electrical Engineering, Quasar Federal Systems, San Diego, USA; 3 Medical Physics, Quasar Federal Systems, San Diego, USA; 4 Neurosurgery, Arrowhead Regional Medical Center, Colton, USA

**Keywords:** speech, neurons, verbalization, visual object naming, shielding, motor mapping, helmet, new technologies in neurosurgery, medical device technology, electromagnetic field

## Abstract

Introduction

Neurologic activity is mediated by electrochemical signaling pathways that generate an electric charge. These electrical signals generate electromagnetic fields (EMF) that have been found to be measurable through magnetoencephalography and induction sensors. These technologies typically rely on expensive shielding via shielded rooms to remove influence from the external environment. We aimed to investigate the effects of a lightweight shielded helmet constructed of Mu-metal and interlaced copper mesh and Mu-metal EMF “channels” on shielding externally mediated EMF when measuring cortically generated EMF during human activity.

Methods

Non-contact, non-invasive, proprietary induction sensors were utilized with a combination of a dual-layer Mu-metal and interlaced copper mesh helmet with sensors placed within EMF channels connected to the helmet. Five human volunteers participated in motor activities, verbalization activities, and visual object naming to evaluate the effectiveness of shielding solely via the helmet and EMF channel construct on generated EMF without placement of individuals within a shielded room. Background data without a subject were obtained.

Results

Differences in fast Fourier transformed data were noted in the background compared to rest and various activities throughout all trials. There were differences in rest phases and activity phases in each individual identifying active measurement of differences in cortically generated EMF during each activity.

Conclusion

It appears that eliminating a Mu-metal shielded room is possible when shielding is isolated to the helmet and EMF channels with induction sensors. The external EMF was appropriately excluded with differences in background data in all trials. During the activity, differences were noted between rest phases and activity phases in all activities noting the discernibility of these induction sensors in measuring cortically generated EMF. Measured activity through motor tapping with changes between 4 and 6 Hz appeared to correlate with previously documented changes during motor activity using these sensors in conjunction with shielded rooms.

## Introduction

Measuring the electromagnetic field (EMF) generated by the cortex of human individuals in vivo has become possible through advances in technology [1-4]. EMF signals generated by the brain are typically less powerful than the surrounding environment [5-7]. Therefore, these signals may be affected and masked by these external signals. To combat this, investigators typically utilized electromagnetic shielding with shielded rooms to evaluate the generated signals [6]. Further, in most circumstances, measuring these signals has only been possible with the usage of superconducting quantum interference devices (SQUIDs) to enhance the sensitivity of the sensors. These SQUIDs require supercooling to generate liquid helium, which results in significant cost and technology [7].

We have found that it was possible to measure these signals in previous studies using proprietary induction sensors (Quasar Federal Systems, San Diego, CA) without the need for these SQUIDs [1-4]. These sensitive, compact induction sensors utilize a 9-volt battery supply and a solenoid coil surrounding a high permeability core to measure low-frequency magnetic fields. Alternative sensor modalities have additionally begun to be investigated in the literature with the investigation of optically pumped magnetometer (OPM) magnetoencephalography (MEG)-based technologies that are not reliant on SQUID; however, these are still under initial investigation [6]. OPM-MEG is not without its limitations, due to reliance on shielding technologies as a result of the need for a static magnetic field that is approximately zero [6]. Therefore, shielded rooms have been required for analysis using OPM-MEG resulting in significant costs and a lack of portability.

In our investigations, the usage of a shielded helmet with an EMF channel using proprietary induction sensors was discussed by Brazdzionis et al. [4]. This helmet and channel structure appeared to provide additional shielding that acted as an adjunct to measure the EMF signals within a shielded room and appeared necessary to optimize EMF evaluation [4]. Further, the utility was such that even as sensors were moved as far as 63 cm away from the subject under investigation, the EMF channel, helmet, and Faraday shield appeared sufficient enough to evaluate a generated EMF of the subject when participating in motor-based activities [3].

Therefore, as this shielding construct appeared to aid in measurements of the generated EMF from significant distances away from the subject, we wished to investigate the efficacy of the induction sensors with the shielded helmet and EMF channel technology on the external environment after removal from the shielded room. In doing so, we wished to evaluate whether the shielded helmet and EMF channel would provide sufficient shielding without a shielded room and outside of a building. External noise and generated signals from the Earth’s magnetic field are thought to provide interference to these sensors without appropriate shielding and would thus impair appropriate data analysis, which would be significantly greater outside a building and shielded room than within it. Therefore, if induction sensors combined with an EMF channel and shielded helmet demonstrate efficacy in shielding, it may be a viable technology to reduce limitations in research related to EMF and MEG technologies that have higher start-up costs due to the need for investments in SQUID-based devices or a shielded room, which can cost hundreds of thousands of US dollars. This may increase access to this technology and its subsequent utilization may increase. Although differing from the induction sensors that are under investigation in this trial, recent studies have begun to investigate non-shielded helmets with OPM-MEG technologies to reduce the need for SQUIDs and with the addition of dynamic field controls and computer suppression of interference to try to minimize associated costs with shielding [6]. Initial studies note an effect of this method with subtraction of noise demonstrating 80% correlation with standard MEG within a shielded room; however, with 80% correlation, there is room for improvement to maximize diagnostic utility [6].

To improve on this novel technology using previously tested sensors, we investigated minimizing shielding by removing the shielded room component and instead relying solely on shielding through a helmet and EMF channel construct. Shielded rooms can cost upwards of hundreds of thousands of dollars, and therefore, the goal was to demonstrate the potential for reduced shielding costs and improved correlation to more expensive shielded rooms beyond that of computational analysis. This may stimulate increased research and evaluation of clinical applications of this EMF-based technology.

## Materials and methods

Institutional review board approval was obtained from Arrowhead Regional Medical Center entitled “In-vivo non-contact remote measurement of neuronal activity” (protocol #: 21-05). A shielded helmet was constructed using dual layers of Mu-metal (MuMETAL®, Magnetic Shield Corporation, Bensenville, IL) separated by 2.5 cm with an inner and outer layer of interlaced copper mesh as demonstrated by Wiginton and Brazdzionis [1-4]. Holes within the helmet were placed with two on the right side and two on the left side. The holes were located over the right temporal region, right frontal region, left frontal region, and left temporal region. Proprietary sensors utilized were model BS-1000 induction sensors (Quasar Federal Systems, San Diego, CA). Each sensor needs a 9 volt (V) supply, consuming 3.3 milliamps (mA). These sensors are designed to evaluate non-direct current, low-frequency magnetic fields between 1 Hz and 30 kHz along a single axis through the use of a solenoid coil surrounding a high-permeability core of 0.2-inch diameter. The sensor is 18 inches long. Detection sensitivity was set at 1.5 pTrms/rtHz at 1 Hz, 0.15 pTrms/rtHz at 10 Hz, 0.025 pTrms/rtHz at 100 Hz, and 0.02 pTrms/rtHz at 1 kHz and above. During the tests, data collection of the sensor response was captured between 1 Hz and 2 kHz. Test configuration utilized an additional gain filter module after the preamplifier, with a 10x gain for amplifying the signal and a 2 kHz low-pass filter for anti-aliasing. Data collection occurred at 5 kilo-samples/second. Sensors were placed within a polyvinyl chloride (PVC) tube surrounded by Mu-metal to act as an EMF channel, were placed within each of the holes, and directed to the desired regions of measurement after securement. As reported by Brazdzionis et al., after ensuring the positive end of the sensors was oriented towards the scalp, sensor Bx was placed in the left temporal region and oriented with the trajectory directed toward the left motor strip, sensor By was placed in the right temporal region and oriented with a trajectory directing toward the left motor strip, sensor Bz was placed in the right frontal region near the right frontal primary motor cortex and oriented with a trajectory directed at the left motor cortex, and sensor B319 was placed in the region of the left motor cortex and directed toward the left motor cortex [4]. Each sensor was placed 4.5 cm from the scalp surface.

Individual tests

Five volunteer adult male subjects all over the age of 18 years were asked to place the helmet on their head and participate in repeated activities to evaluate generated EMF from each subject’s cortical structures during designated activities. Subjects were placed within a fabric tent with the shielded helmet placed on their heads. The helmet was supported via a line and pulley system with suspension from above and support from stands and tripods below to eliminate motion, such as from the wind, during testing as small motions can induce magnetic noise in induction sensors due to sensor motion within the direct current (DC) Earth field. Mu-metal shielded room and Faraday cage were not utilized during testing and instead the shielding for analysis came strictly from the helmet and EMF channel construct. Subjects were asked to participate in several activities using a modification of the reproducible synchronizing stimulus protocol described by Brazdzionis et al. with substitutions of the designated motor activity for the action activity under investigation [3]. This protocol consisted of 30 seconds of resting activity wherein the subject was informed to attempt to have an absence of thought and activity, 60 seconds of investigated activity, and 30 seconds of resting activity. During each activity, a metronome was set at 2 Hz for consistency of stimulus and synchronization of timing. Prior to testing with a subject, an initial baseline test was conducted using the helmet with EMF channels in place suspended within the tent without a subject to evaluate the baseline EMF evaluated by the sensors. The metronome was active at 2 Hz as a control during this test.

Subjects were asked to participate in the following activities: right-hand tapping at 2 Hz with the metronome, language activities through verbalization of the English alphabet with one letter per metronome click (120 beats/min), and visual object naming using a deck of cards to name as many cards as possible within the 60-second investigated period. During visual object naming, subjects were asked to minimize motor movement when moving a playing card from the right hand to the left hand after naming a card; however, motor activity was required during the activity. During all trials, the generated frequency and waves were analyzed in real time to evaluate for motion artifact, and if motion artifact was present, the test was repeated from the beginning. Right-hand tapping occurred similarly to protocols described by Wiginton and Brazdzionis [2,3]. During right-hand tapping activities, the subject was seated on a chair with the right arm supported while participating in 30 seconds of rest followed by right-hand tapping at 2 Hz (1 tap per 0.5 seconds) for 60 seconds followed by 30 seconds of rest inactivity. The wrist remained fixed on the armrest while the tips of the fingers were elevated upwards and brought back down to the arm rest to complete a single tap. During this activity, subjects were asked to keep their eyes closed to minimize visual stimulus.

During verbalization of the English alphabet, subjects remained seated with the arms, sensors, and helmet supported and were asked to recite the alphabet in its standard order at a rate of one letter per 0.5 seconds during the 60-second activity phase of the experiment; the alphabet was repeated from beginning to end throughout the duration of the activity phase. The preceding 30-second rest period and the post-30-second rest period in relation to activity were maintained. Subjects were asked to keep their eyes closed during this trial to minimize visual stimulation.

Finally, during visual object naming, subjects were again seated and supported in the same position. Participation was 30 seconds of rest followed by 60 seconds of visual object naming followed by 30 seconds of rest for a total of 120 seconds. The subject was asked to name the card and suit of a playing card in the right hand and pass it to the left hand with minimal movement. Subjects were asked to name as many cards as possible during the 60-second activity period. The metronome was again set at 2 Hz for consistency although the timing was not utilized during this trial.

Data analysis and technical specifications

A 16-bit National Instruments Data Acquisition Card and LabVIEW software (National Instruments Corporation, Austin, TX) were utilized as described by Wiginton et al. [1]. Data were analyzed using Igor® Pro 8 software (Wavemetrics Inc., Lake Oswego, OR), with fast Fourier transform (FFT) frequency-domain data analyzed with cumulative composite voltages in bins versus frequency of the middle 20-second blocks similar to data analysis completed by Wiginton et al. [1]. For all trial’s rest data, the selected 20-second bin under investigation was between five and 25 seconds. During evaluated activity data, the selected region of analysis was data observed between 50 and 70 seconds.

## Results

Baseline testing without a subject

**Figure 1:**
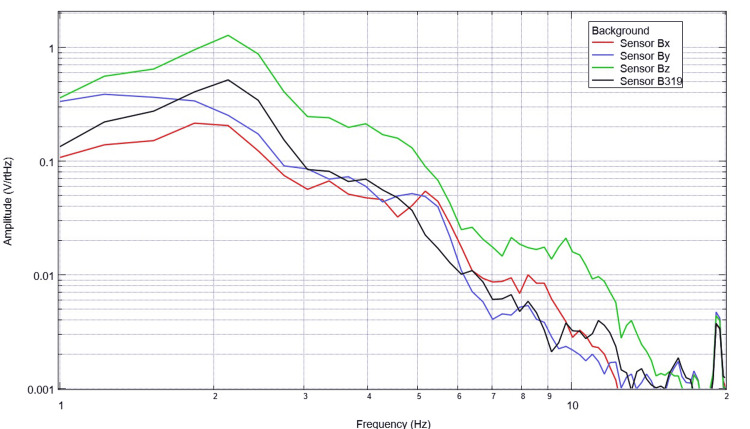
Baseline background data from outdoor testing Baseline background data are demonstrated without a subject participating in activities using logarithmic scales. Amplitude is related to the number of points in the domain signal due to fast Fourier transformed data. Sensor Bx is indicated by the red line, sensor By by blue, sensory Bz by green, and sensor B319 by black. Hz: Hertz; V/rtHz: voltage divided by the square root of Hertz.

Initial baseline EMF data were evaluated without a subject and while outdoors within the tent and suspension construct. Initial baseline waveforms after FFT are seen below in Figure 1.

Measurement of generated EMF through motor activities

**Figure 2:**
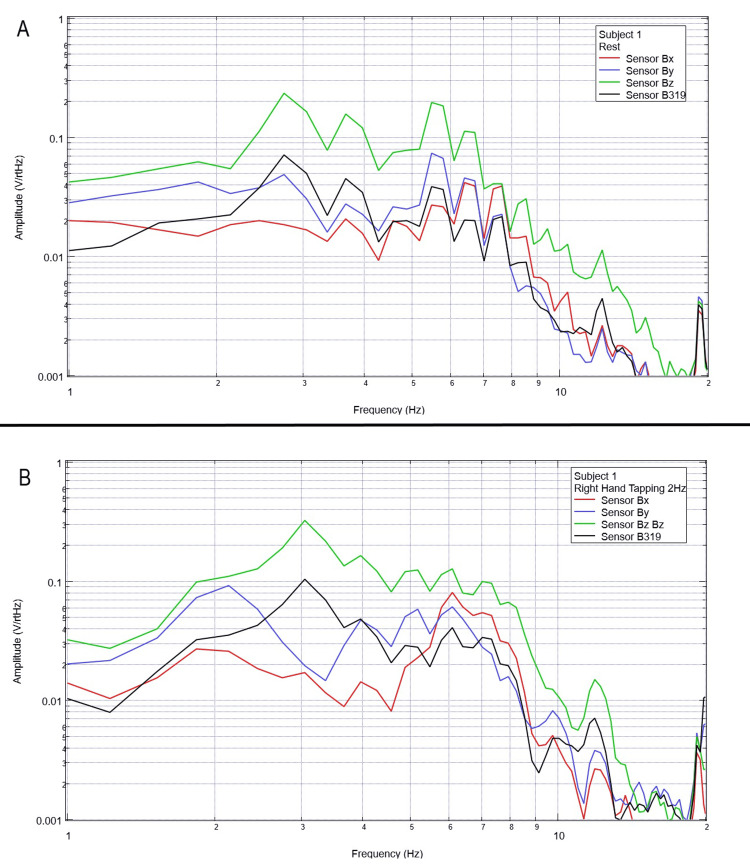
Subject 1 rest phase and right-hand tapping at 2 Hz Baseline rest data are demonstrated in panel A identifying baseline measured EMF activity for subject 1 prior to tapping while panel B identifies measured EMF activity during tapping activities for subject 1. Plots utilize logarithmic scales. Amplitude is related to the number of points in the domain signal due to fast Fourier transformed data. Sensor Bx is indicated by the red line, sensor By by blue, sensory Bz by green, and sensor B319 by black. Hz: Hertz; V/rtHz: voltage divided by the square root of Hertz; EMF: electromagnetic field.

**Figure 3:**
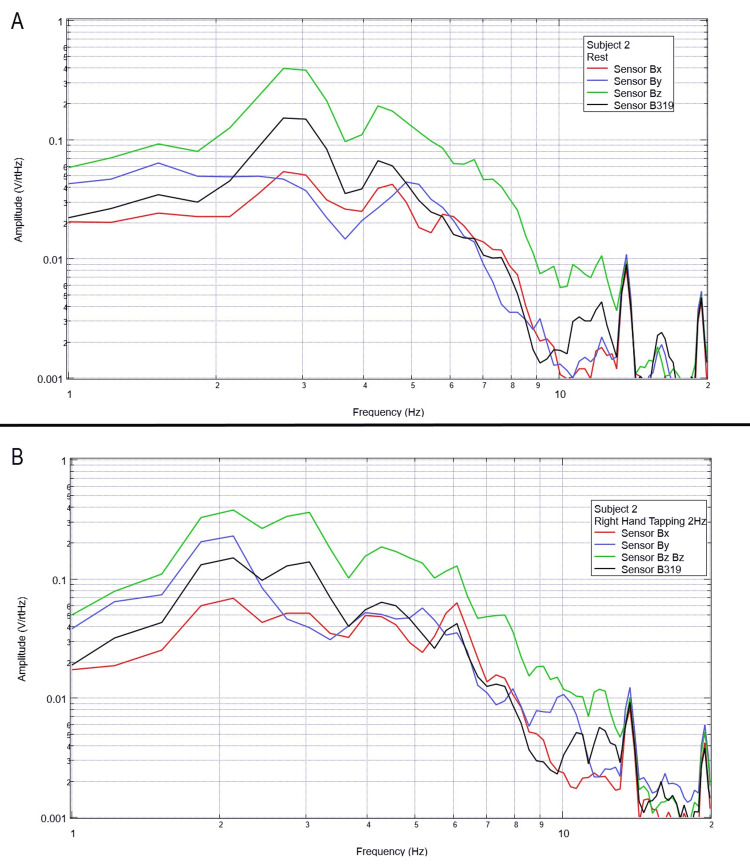
Subject 2 rest phase and right-hand tapping at 2 Hz Baseline rest data are demonstrated in panel A identifying baseline measured EMF activity for subject 2 prior to tapping while panel B identifies measured EMF activity during tapping activities for subject 2. Plots utilize logarithmic scales. Amplitude is related to the number of points in the domain signal due to fast Fourier transformed data. Sensor Bx is indicated by the red line, sensor By by blue, sensory Bz by green, and sensor B319 by black. Hz: Hertz; V/rtHz: voltage divided by the square root of Hertz; EMF: electromagnetic field.

**Figure 4:**
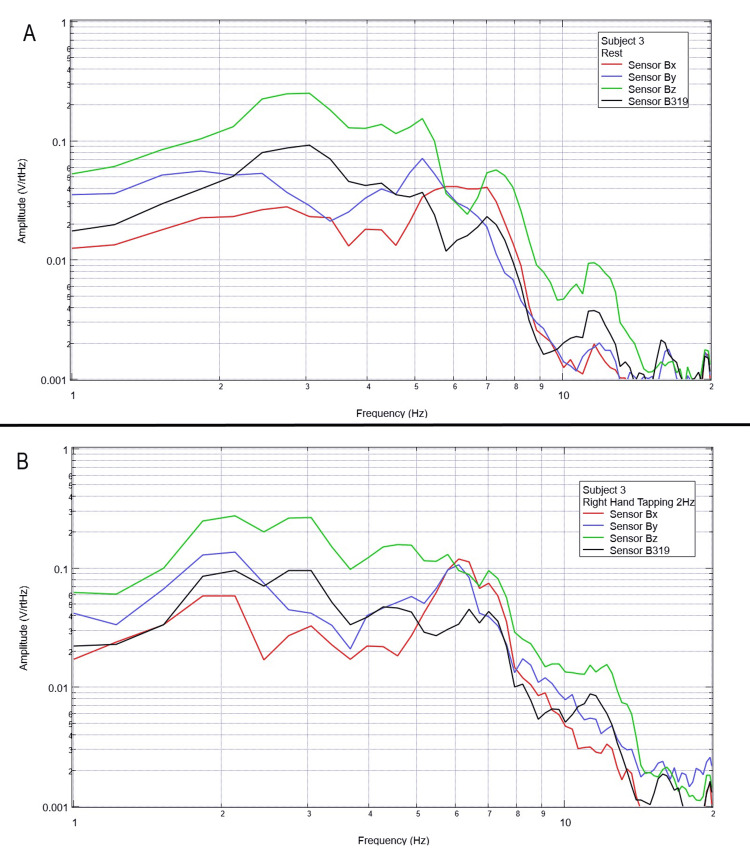
Subject 3 rest phase and right-hand tapping at 2 Hz Baseline rest data are demonstrated in panel A identifying baseline measured EMF activity for subject 3 prior to tapping while panel B identifies measured EMF activity during tapping activities for subject 3. Plots utilize logarithmic scales. Amplitude is related to the number of points in the domain signal due to fast Fourier transformed data. Sensor Bx is indicated by the red line, sensor By by blue, sensory Bz by green, and sensor B319 by black. Hz: Hertz; V/rtHz: voltage divided by the square root of Hertz; EMF: electromagnetic field.

**Figure 5:**
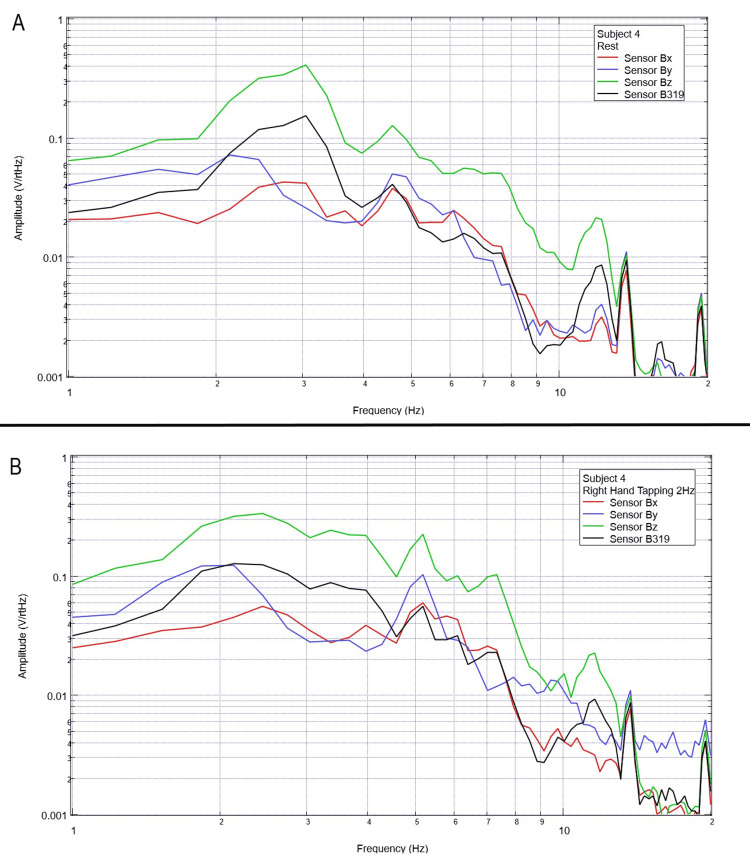
Subject 4 rest phase and right-hand tapping at 2 Hz Baseline rest data are demonstrated in panel A identifying baseline measured EMF activity for subject 4 prior to tapping while panel B identifies measured EMF activity during tapping activities for subject 4. Plots utilize logarithmic scales. Amplitude is related to the number of points in the domain signal due to fast Fourier transformed data. Sensor Bx is indicated by the red line, sensor By by blue, sensory Bz by green, and sensor B319 by black. Hz: Hertz; V/rtHz: voltage divided by the square root of Hertz; EMF: electromagnetic field.

**Figure 6:**
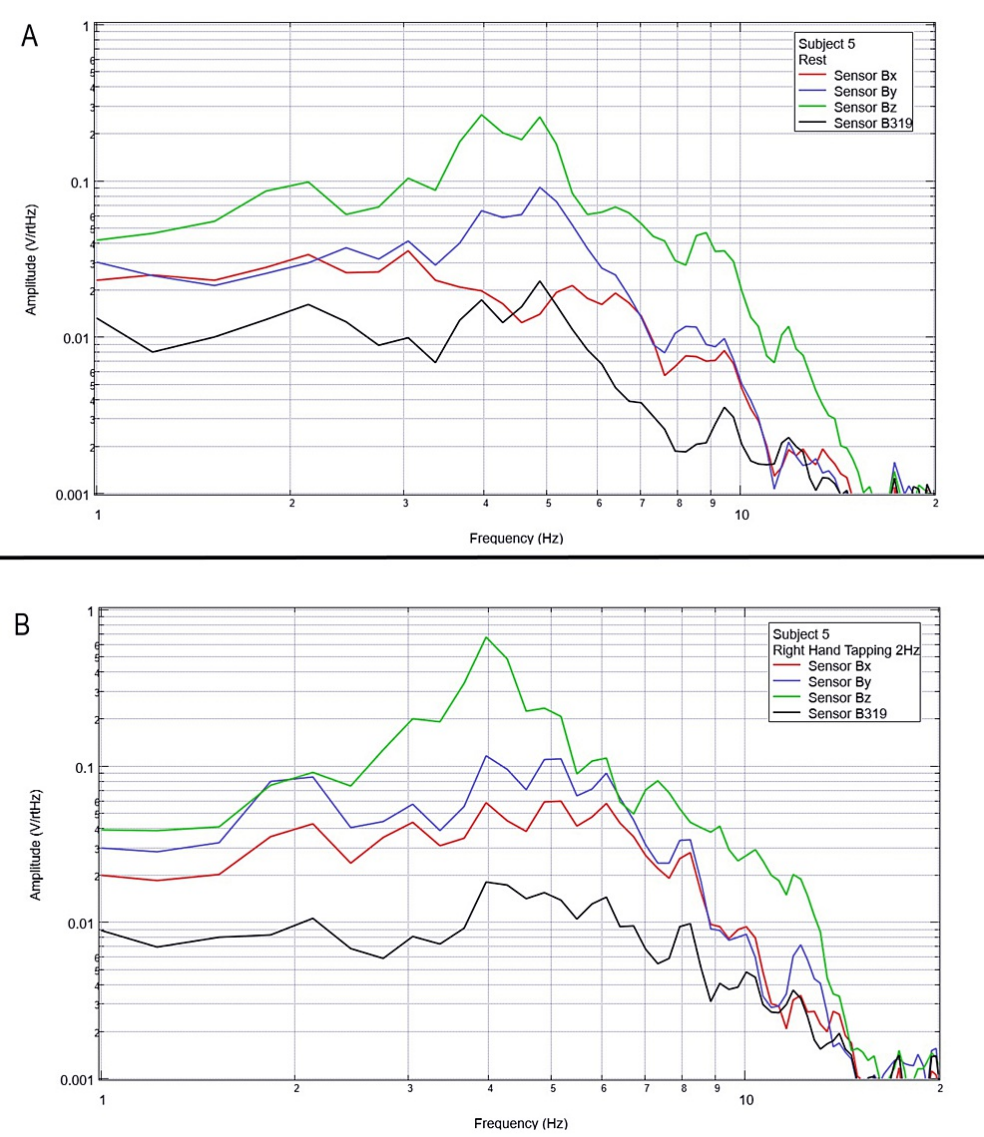
Subject 5 rest phase and right-hand tapping at 2 Hz Baseline rest data are demonstrated in panel A identifying baseline measured EMF activity for subject 5 prior to tapping while panel B identifies measured EMF activity during tapping activities for subject 5. Plots utilize logarithmic scales. Amplitude is related to the number of points in the domain signal due to fast Fourier transformed data. Sensor Bx is indicated by the red line, sensor By by blue, sensory Bz by green, and sensor B319 by black. Hz: Hertz; V/rtHz: voltage divided by the square root of Hertz; EMF: electromagnetic field.

Each of the five subjects was instructed to participate in a repeated motor activity through right-hand tapping at a rate of 2 Hz synchronized by a metronome with a preceding 30 seconds of rest period and with a subsequent 30 seconds of rest period after the activity. Baseline rest and tapping data are identified below for each subject in plots of FFT data in Figures 2-6.

When evaluating baseline EMF seen in Figure 1 compared to Figures 2-6 in both rest phases and tapping phases, the evaluated EMF waves appear different from baseline with increased activity between 4 and 6 Hz in all with differing peak morphologies compared to non-subject trials. Further, when evaluating Figures 2-6 for differences between rest phases (panel A), each figure differed from tapping phases (panel B) for all subjects. These results reveal a discernable difference despite only using a shielded helmet and EMF channel construct (without the use of a shielded room). Baseline external EMF measured by the helmet and EMF channel construct without a subject compared to baseline rest data as well as differences between baseline rest data and motor activity in each subject showed no need for a shielded room.

Measurement of generated EMF through verbalization activities

**Figure 7:**
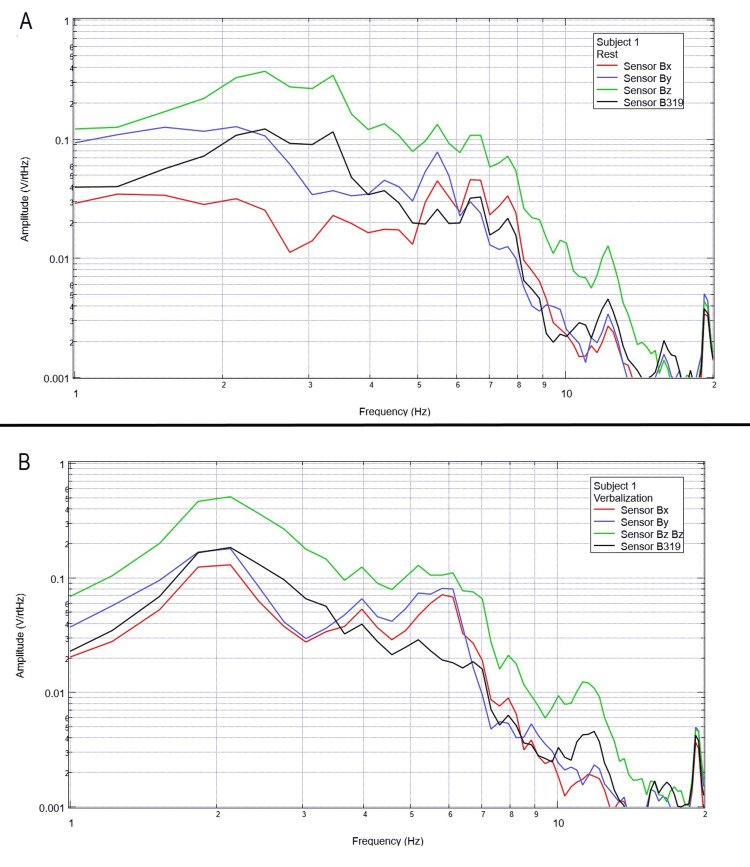
Subject 1 rest phase and verbalization Baseline rest data are demonstrated in panel A identifying baseline measured EMF activity for subject 1 prior to verbalization while panel B identifies measured EMF activity during verbalization for subject 1. Plots utilize logarithmic scales. Amplitude is related to the number of points in the domain signal due to fast Fourier transformed data. Sensor Bx is indicated by the red line, sensor By by blue, sensory Bz by green, and sensor B319 by black. Hz: Hertz; V/rtHz: voltage divided by the square root of Hertz; EMF: electromagnetic field.

**Figure 8:**
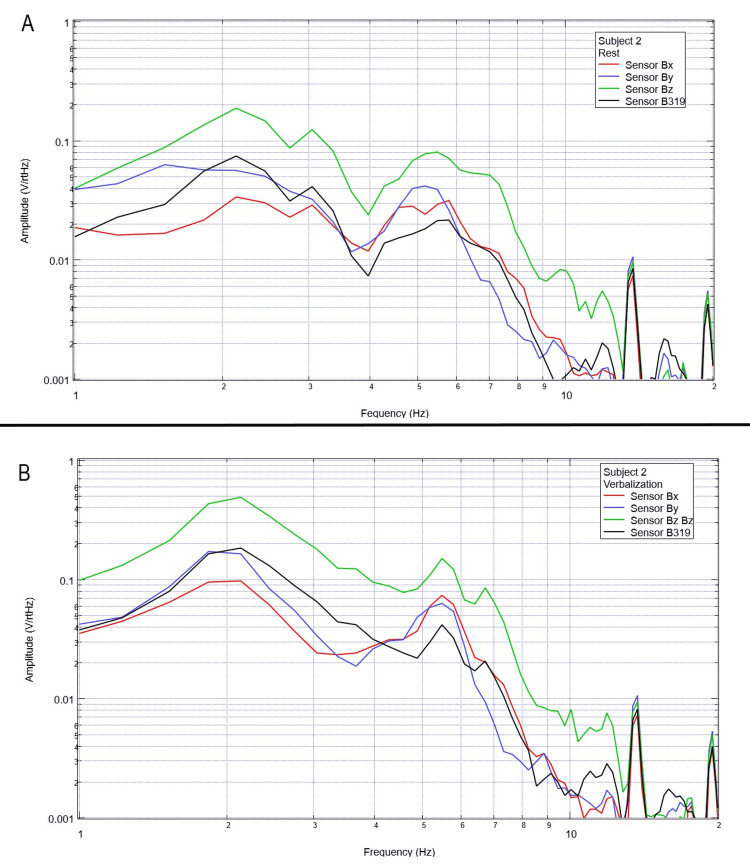
Subject 2 rest phase and verbalization Baseline rest data are demonstrated in panel A identifying baseline measured EMF activity for subject 2 prior to verbalization while panel B identifies measured EMF activity during verbalization for subject 2. Plots utilize logarithmic scales. Amplitude is related to the number of points in the domain signal due to fast Fourier transformed data. Sensor Bx is indicated by the red line, sensor By by blue, sensory Bz by green, and sensor B319 by black. Hz: Hertz; V/rtHz: voltage divided by the square root of Hertz; EMF: electromagnetic field.

**Figure 9:**
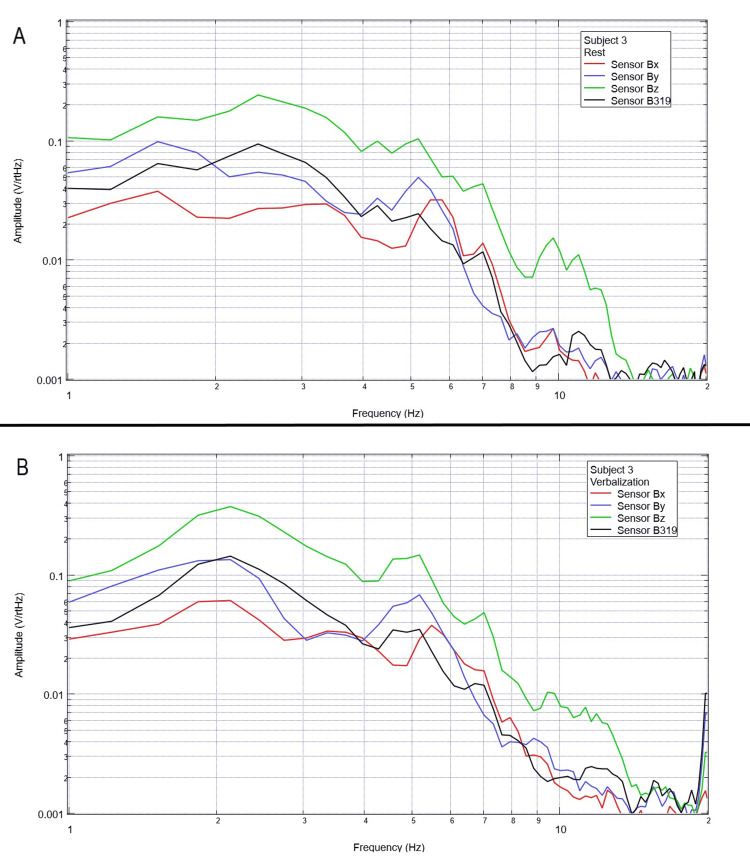
Subject 3 rest phase and verbalization Baseline rest data are demonstrated in panel A identifying baseline measured EMF activity for subject 3 prior to verbalization while panel B identifies measured EMF activity during verbalization for subject 3. Plots utilize logarithmic scales. Amplitude is related to the number of points in the domain signal due to fast Fourier transformed data. Sensor Bx is indicated by the red line, sensor By by blue, sensory Bz by green, and sensor B319 by black. Hz: Hertz; V/rtHz: voltage divided by the square root of Hertz; EMF: electromagnetic field.

**Figure 10:**
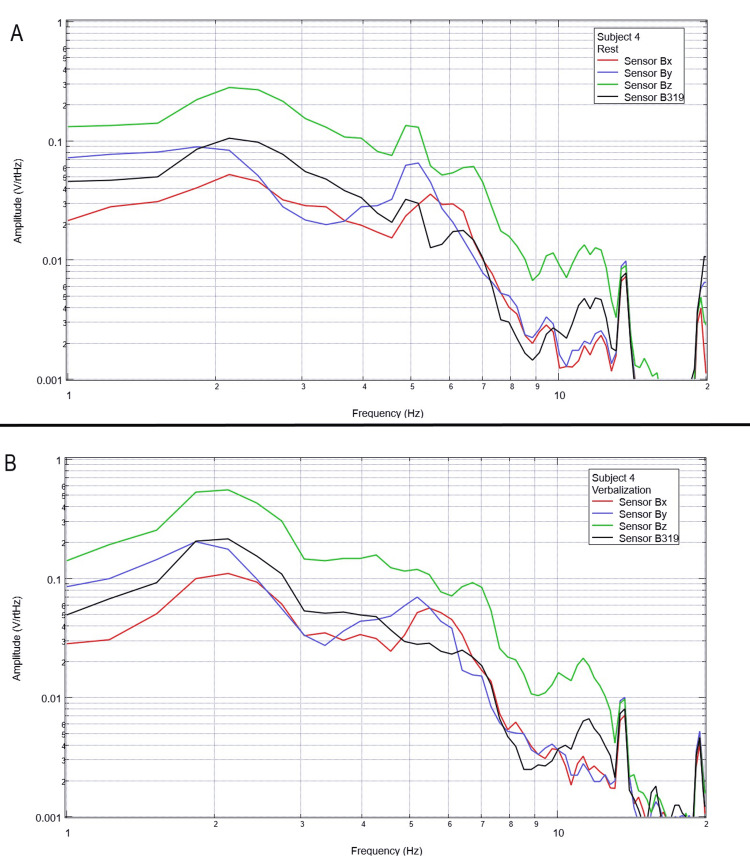
Subject 4 rest phase and verbalization Baseline rest data are demonstrated in panel A identifying baseline measured EMF activity for subject 4 prior to verbalization while panel B identifies measured EMF activity during verbalization for subject 4. Plots utilize logarithmic scales. Amplitude is related to the number of points in the domain signal due to fast Fourier transformed data. Sensor Bx is indicated by the red line, sensor By by blue, sensory Bz by green, and sensor B319 by black. Hz: Hertz; V/rtHz: voltage divided by the square root of Hertz; EMF: electromagnetic field.

**Figure 11:**
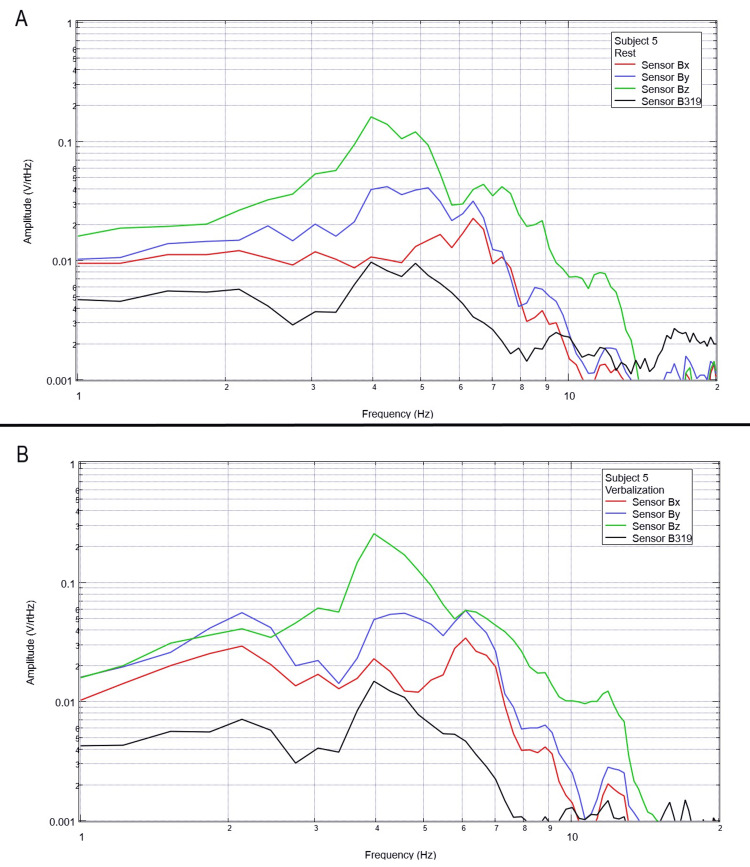
Subject 5 rest phase and verbalization Baseline rest data are demonstrated in panel A identifying baseline measured EMF activity for subject 5 prior to verbalization while panel B identifies measured EMF activity during verbalization for subject 5. Plots utilize logarithmic scales. Amplitude is related to the number of points in the domain signal due to fast Fourier transformed data. Sensor Bx is indicated by the red line, sensor By by blue, sensory Bz by green, and sensor B319 by black. Hz: Hertz; V/rtHz: voltage divided by the square root of Hertz; EMF: electromagnetic field.

Each subject was again instructed to don the shielded helmet and EMF channel construct while within the tent but without the usage of a shielded room. Subjects were each asked to recite the English alphabet (one letter per 0.5 seconds as synchronized with the metronome). Rest phase data and alphabet verbalization data are seen below in Figures 7-11.

Baseline resting data seen in each panel A of Figures 7-11 is again noted to be different than baseline EMF evaluated without a subject seen in Figure 1. This appears to confirm the efficacy of the EMF helmet and EMF channel construct in evaluating a human subject's generated EMF without the need for a shielded room. Further, when evaluating verbalization activities seen in panel B of Figures 7-11, there are noted differences in morphology between the rest phase for each subject and the verbalization phase of each subject. These differences vary from subject to subject; however, they appear most frequently between 5 and 6 Hz. Overall there seems to be a loss of an additional peak also described as “flattening” of the curve between 5 and 6 Hz when comparing baseline resting activity to verbalization activity with the “flattening” occurring during the verbalization phases. Due to FFT effects wherein more frequently identified amplitudes have higher frequencies, this increased activity functionally changes the morphology of these curves by either “increasing peaks” or “flattening” curves. Therefore, these changes within curves demonstrate shifts in the activity of measured EMF during verbalization.

Measurement of generated EMF through visual object naming activities

**Figure 12:**
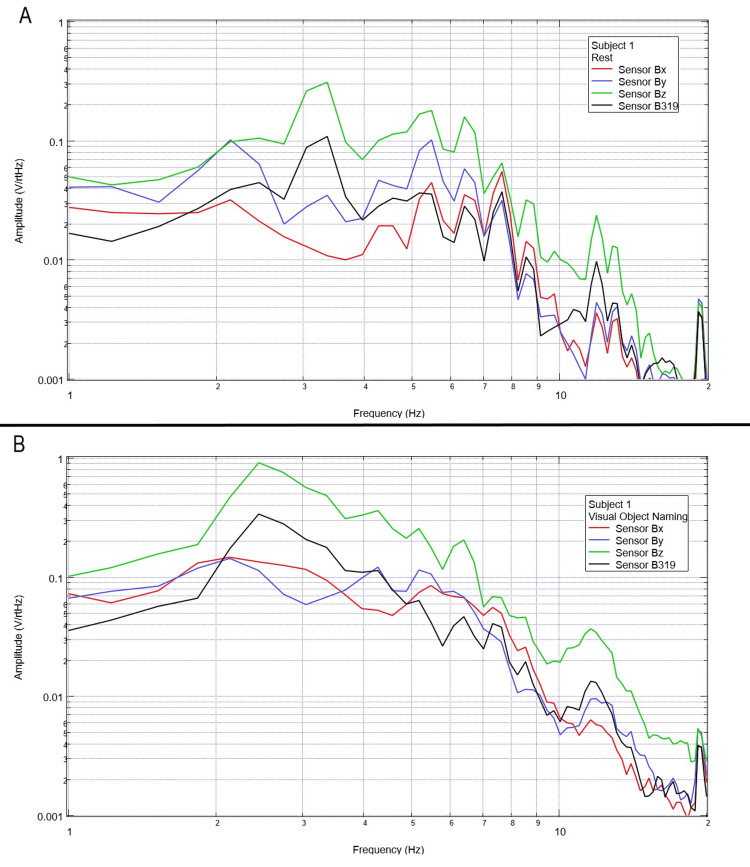
Subject 1 rest phase and visual object naming Baseline rest data are demonstrated in panel A identifying baseline measured EMF activity for subject 1 prior to visual object naming while panel B identifies measured EMF activity during visual object naming for subject 1. Plots utilize logarithmic scales. Amplitude is related to the number of points in the domain signal due to fast Fourier transformed data. Sensor Bx is indicated by the red line, sensor By by blue, sensory Bz by green, and sensor B319 by black. Hz: Hertz; V/rtHz: voltage divided by the square root of Hertz; EMF: electromagnetic field.

**Figure 13:**
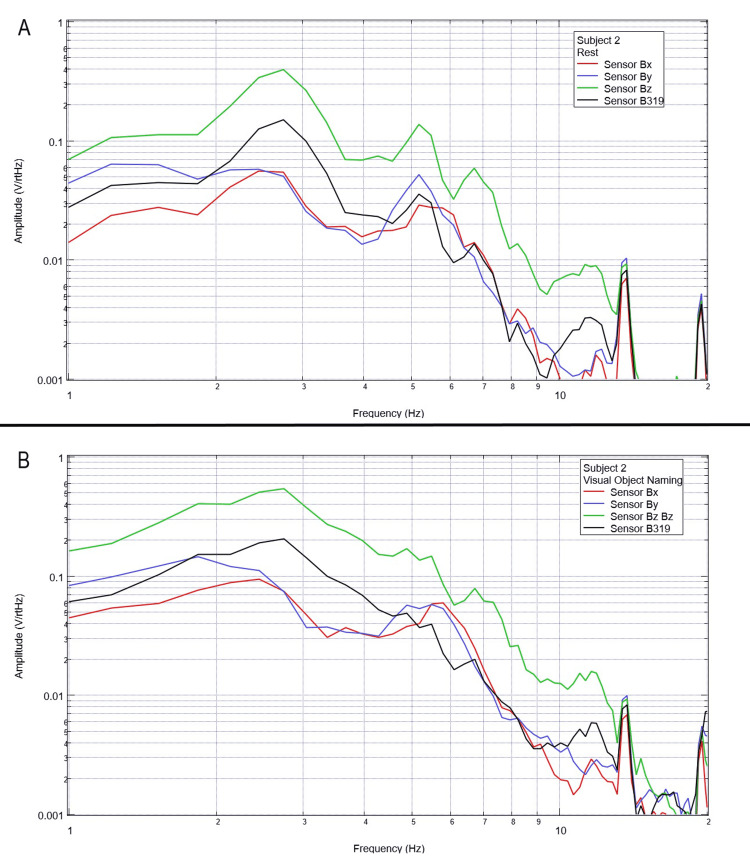
Subject 2 rest phase and visual object naming Baseline rest data are demonstrated in panel A identifying baseline measured EMF activity for subject 2 prior to visual object naming while panel B identifies measured EMF activity during visual object naming for subject 2. Plots utilize logarithmic scales. Amplitude is related to the number of points in the domain signal due to fast Fourier transformed data. Sensor Bx is indicated by the red line, sensor By by blue, sensory Bz by green, and sensor B319 by black. Hz: Hertz; V/rtHz: voltage divided by the square root of Hertz; EMF: electromagnetic field.

**Figure 14:**
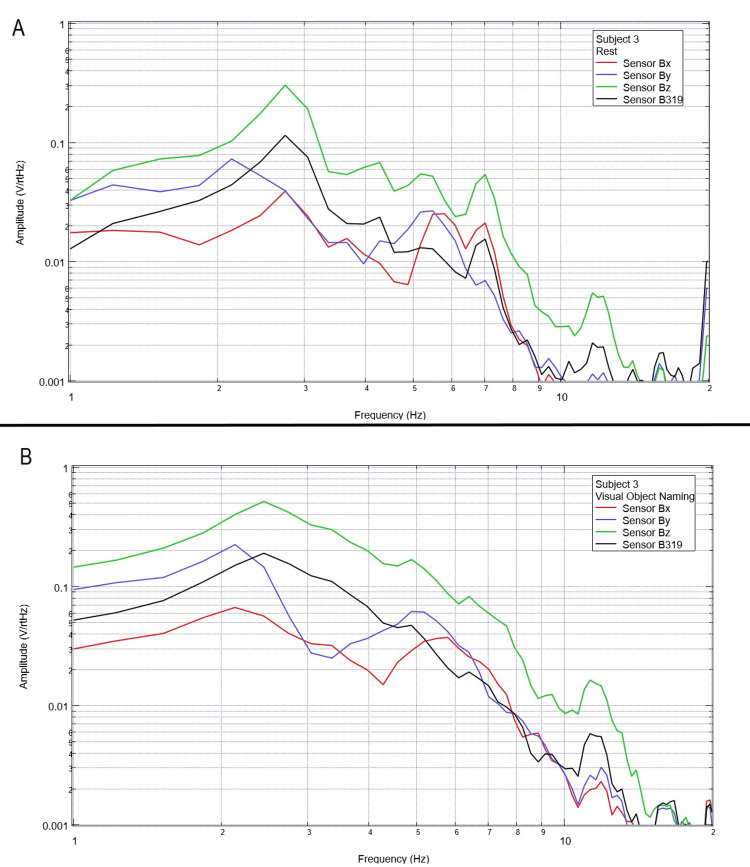
Subject 3 rest phase and visual object naming Baseline rest data are demonstrated in panel A identifying baseline measured EMF activity for subject 3 prior to visual object naming while panel B identifies measured EMF activity during visual object naming for subject 3. Plots utilize logarithmic scales. Amplitude is related to the number of points in the domain signal due to fast Fourier transformed data. Sensor Bx is indicated by the red line, sensor By by blue, sensory Bz by green, and sensor B319 by black. Hz: Hertz; V/rtHz: voltage divided by the square root of Hertz; EMF: electromagnetic field.

**Figure 15:**
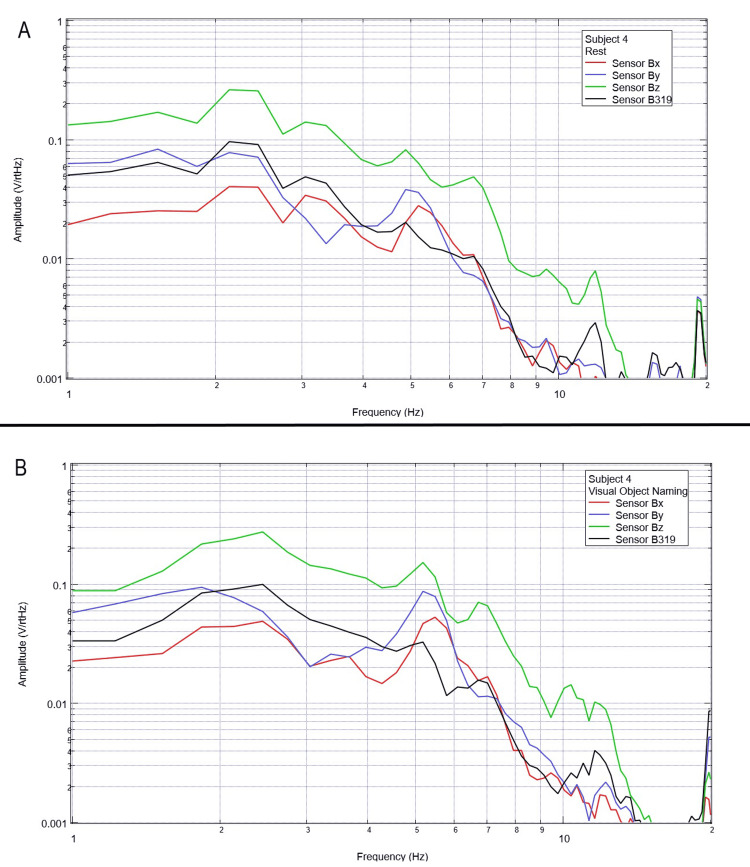
Subject 4 rest phase and visual object naming Baseline rest data are demonstrated in panel A identifying baseline measured EMF activity for subject 4 prior to visual object naming while panel B identifies measured EMF activity during visual object naming for subject 4. Plots utilize logarithmic scales. Amplitude is related to the number of points in the domain signal due to fast Fourier transformed data. Sensor Bx is indicated by the red line, sensor By by blue, sensory Bz by green, and sensor B319 by black. Hz: Hertz; V/rtHz: voltage divided by the square root of Hertz; EMF: electromagnetic field.

**Figure 16:**
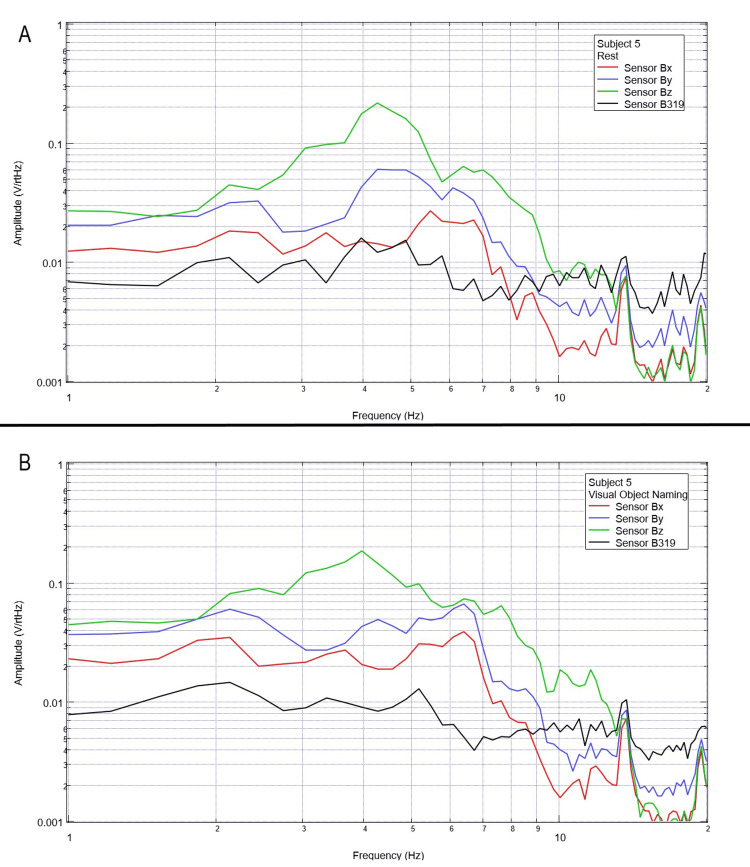
Subject 5 rest phase and visual object naming Baseline rest data are demonstrated in panel A identifying baseline measured EMF activity for subject 5 prior to visual object naming while panel B identifies measured EMF activity during visual object naming for subject 5. Plots utilize logarithmic scales. Amplitude is related to the number of points in the domain signal due to fast Fourier transformed data. Sensor Bx is indicated by the red line, sensor By by blue, sensory Bz by green, and sensor B319 by black. Hz: Hertz; V/rtHz: voltage divided by the square root of Hertz; EMF: electromagnetic field.

Final testing occurred to evaluate the effects of compound activity, particularly visual object naming. Subjects were instructed to visualize and subsequently name verbally as many playing cards as possible from a standard deck of playing cards while moving the cards with minimal movement from the right hand to the left hand. Rest phase data between five and 25 seconds and activity phase data between 50 and 70 seconds were analyzed using FFT and are plotted below in Figures 12-16.

In our final trials on visual object naming, changes between baseline EMF without a subject (Figure 1) were again different from baseline rest and activity data seen in all subjects (Figures 12-16). This further supports the efficacy of the EMF channel and helmet construct to adequately shield from the unwanted electromagnetic activity without the need for a full electromagnetically shielded enclosure. Further analysis investigating differences in rest data in the “A” panels of Figures 12-16 compared to the visual object naming activity “B” panels shows morphology differences. These morphology changes appear to occur in a wider range of frequencies compared to previous investigations occurring between 3 and 6 Hz.

## Discussion

This project evaluated the feasibility of measuring an EMF using induction sensors with a Mu-metal shielded helmet and EMF channels while outside of a shielded room. Multiple subjects and differing cortical activities were utilized to evaluate for differences, if any, in morphologies comparing rest, motor activity, and visual object naming. The results indicate that each activity had demonstrably different morphologies compared to baseline rest activity immediately prior to each activity. Further, the measured EMF at rest and during each activity appeared to differ in each of the five subjects.

Due to the effects of the FFT function, changes represent summative repeated measurements in activity. Therefore, increases or changes in activity represent a repeated subset of measured EMF activity during the evaluated period. As each period measured was a 20-second selected bin, the summative total temporal period of measurement did not differ. Further, these changes in summative repeated activity likely represent regions of the brain more actively generating excitatory and inhibitory postsynaptic potentials to create a temporal summation of activity and electrical charges generating an EMF. These changes in activity, therefore, represent areas of cortical activity and the above differences in the FFT curves identify the effectiveness of this prototype helmet with inductive sensors and EMF channels in measuring EMF within the conical region of sensing for each sensor without the need for a shielded room.

Previous studies have investigated induction sensors on measured EMF in human subjects participating in motor activities, motor imagery, and emotional thought [2,3]. Therefore, after establishing baseline background data without a subject (Figure 1), the initial investigation commenced by utilizing right-hand tapping exercises as demonstrated in Figures 2-5 [2,3]. Noted differences were seen in rest data compared to background (non-subject data) as well as comparing rest, background, and motor activities. These differences in morphology and overall FFT curves identify efficacy with this shielding modality. Furthermore, these initial studies had not investigated speech through verbalization or visual object naming [1-4]. As visual object naming is a well-defined cortical pathway, this led to selection for evaluation [8]. As seen in Figures 12-16, there were noted differences between rest activity and visual object naming activity evaluated by the sensors. This demonstrates differences in activity related to the active visual, motor, and speech pathways as seen in the differences between 3 and 6 Hz. Similarly, trials in verbalization through the recitation of the English alphabet, which rely on verbal memory and coordination of motor function (Figures 7-11), also demonstrated morphologic characteristics in all five subjects. This supports the efficacy of this prototype in measuring different types of EMF when the subjects are not placed within a shielded room.

Effects of micromotion on induction sensors were previously investigated and evaluated without significant effects on output and measured EMF [3]. However, the possibility of large-scale macromotion masking differing measured EMF or changes in the measured EMF due to cortical activity resulting in that macromotion was considered. Therefore, the helmet was suspended from a frame within the tent and sensors were supported to limit motion. During each test, real-time analysis was conducted to evaluate waveform morphologies and alterations in the waveforms due to gyroscopic output seen on the monitor. Tests were halted and repeated if a significant motion was seen.

The potential benefits from limiting shielding to the helmet and EMF channel include potential cost savings due to the usual high costs in the creation of a shielded room. Further opportunity costs for the space required to contain the shielded room are also considered, in that the shielded room takes up a fixed clinical or research space that traditionally is not utilized for other purposes. As this compact, lightweight helmet and EMF channel device appear to appropriately shield, the decreased opportunity costs could promote more easy clinical usage as well as increase research opportunities, as individuals would not have to retrofit their own spaces to conduct evaluations. Since the shielding is constrained to the helmet itself, there is increased portability allowing for transport between locations. In future studies, increased clinical usage through the use of a point of care type helmet or device could be feasible due to this portability. This reduces risks related to the transport of patients for imaging, and as shielding was appropriate in an external environment, it may potentially be beneficial in spaces with other technologies present, such as an inpatient room. Further, portability may also promote increased ease of access to allow for direct comparison with altering modalities such as OPM-MEGs or other MEG devices.

Although OPM-MEG devices and other sensor modalities were not utilized in this trial, future studies may attempt to utilize this two-layer Mu-metal shielding with an interlaced copper mesh design and integrate it into alternate sensor modalities to evaluate efficacy in shielding external EMF with these different sensor types during EMF evaluation. It appears that the shielded helmet and EMF channel construct act in a summative fashion for appropriate shielding and measuring. Furthermore, it appears the helmet is a necessary component for shielding as seen in previous trials [4]. The EMF was not appropriately evaluated when just an EMF channel was utilized, even when the sensors were brought within the appropriate effective sensor range [4]. Finally, it was also demonstrated that the combined EMF channel and helmet combination was effective within a shielded room to measure a subject as far as 63 cm away, likely due to effects secondary to appropriate shielding of external EMF or reflection of targeted EMF [4].

During this trial, it was noted that despite nearby moving vehicles and flying aircraft within the proximity of the sensors, there was no interference or artifact seen in real time while the subjects were being evaluated. During other such experiments in this lab, notably by Carson et al., even though mouse hippocampal cells were appropriately evaluated within Mu-metal tubes with air gaps, there was intermittent real-time evidence of outside interference presumably from moving vehicles, lab equipment, large electronics, or aircraft overhead [9]. It is hypothesized that the dual-layer Mu-metal helmet with interlaced copper mesh shielding combined with EMF channels appeared to provide appropriate shielding to minimize these potential interferences for our trials within the cone of measurement of each induction sensor.

In evaluating quantitative metrics, there are noted similarities within this trial compared to previous studies investigating these induction sensors [1-4]. Changes in amplitude are seen within all evaluated frequencies between all regions of testing between 2 and 10 Hz, which may be the target of investigation due to various regions of activation of neuronal activity. However, most significantly, a common theme between these studies is that there are changes in the morphology of the FFT curves between approximately 4 and 6 Hz. This is seen again during each trial in this study. Within this specific study, an added region of change was seen between 3 and 4 Hz during visual object naming. As visual object naming was not previously studied using these sensor modalities, this may be a region of interest in the investigation of visual or speech pathways. Further, as the 4-6 Hz region of change is common between each of these studies, this may be an area of specific interest in future studies as a target for cortical EMF as this appears to be the region with the most change correlated to activity. Finally, as these areas of morphologic change are similar in this trial without a shielded room compared to the previous trials using a Faraday shield, this indicates the overall efficacy of a shielded helmet and EMF channel construct as an effective method of shielding external EMF to allow for appropriate and reliable signal measurements using induction sensors.

Limitations

This project was conducted with a small sample size and was conducted on healthy greater than 18-year-old males, which may not represent the overall population. Further, these induction sensors were not compared to MEG technologies to identify temporal relationships between peaks or evaluate signals with known data. Additional studies may be needed to utilize MEG technology and compare it with this novel induction sensor and helmet construct to assist in defining “normal” values for clinical applications. Further studies may also utilize known OPM-MEG technologies with integration into the shielded helmet to see if this shielding is sufficient with alternative sensor modalities.

## Conclusions

Through evaluation of repetitive motor activities, alphabet verbalization, and visual object naming, it appears that a dual-layer helmet constructed with two sheets of Mu-metal and interlaced copper mesh combined with an EMF channel made of Mu-metal are effective methods of shielding external EMF when measuring the brain's intrinsically generated EMF. It appears that this method is durable enough to promote adequate and appropriate shielding without the need for a Mu-metal shielded room or Faraday shield. This may lead to increased research into EMF and MEG technologies due to point of care ability and increased portability of such devices leading to clinical applications and correlation.

## References

[1] Wiginton J 4th, Brazdzionis J, Patchana T, Hung J, Zhang Y, Miulli DE (2022). Novel method of electromagnetic field measurements of the human brain. Cureus.

[2] Wiginton J, Brazdzionis J, Patchana T, Savla P, Hung J, Zhang Y, Miulli DE (2022). Measuring electromagnetic field activity generated by neurons in vivo by humans with thoughts of repetitive motor activities and emotional thoughts. Cureus.

[3] Brazdzionis J, Wiginton J, Patchana T, Savla P, Hung J, Zhang Y, Miulli DE (2022). Evaluating the intrinsic electromagnetic field generated by neurons from repetitive motor activities in humans with a non-contact non-invasive electromagnetic helmet. Cureus.

[4] Brazdzionis J, Wiginton J, Patchana T, Savla P, Hung J, Zhang Y, Miulli DE (2022). Measuring the electromagnetic field of the human brain at a distance using a shielded electromagnetic field channel. Cureus.

[5] Hales CG (2016). The origins of the brain’s endogenous electromagnetic field and its relationship to provision of consciousness. Biophysics of Consciousness: A Foundational Approach.

[6] Hill RM, Devasagayam J, Holmes N (2022). Using OPM-MEG in contrasting magnetic environments. Neuroimage.

[7] Singh SP (2014). Magnetoencephalography: basic principles. Ann Indian Acad Neurol.

[8] Negwer C, Ille S, Hauck T (2017). Visualization of subcortical language pathways by diffusion tensor imaging fiber tracking based on rTMS language mapping. Brain Imaging Behav.

[9] Carson TA, Ghanchi H, Toor H, Majeed G, Wiginton JG 4th, Zhang Y, Miulli DE (2018). Novel method of non-contact remote measurement of neuronal electrical activity. Cureus.

